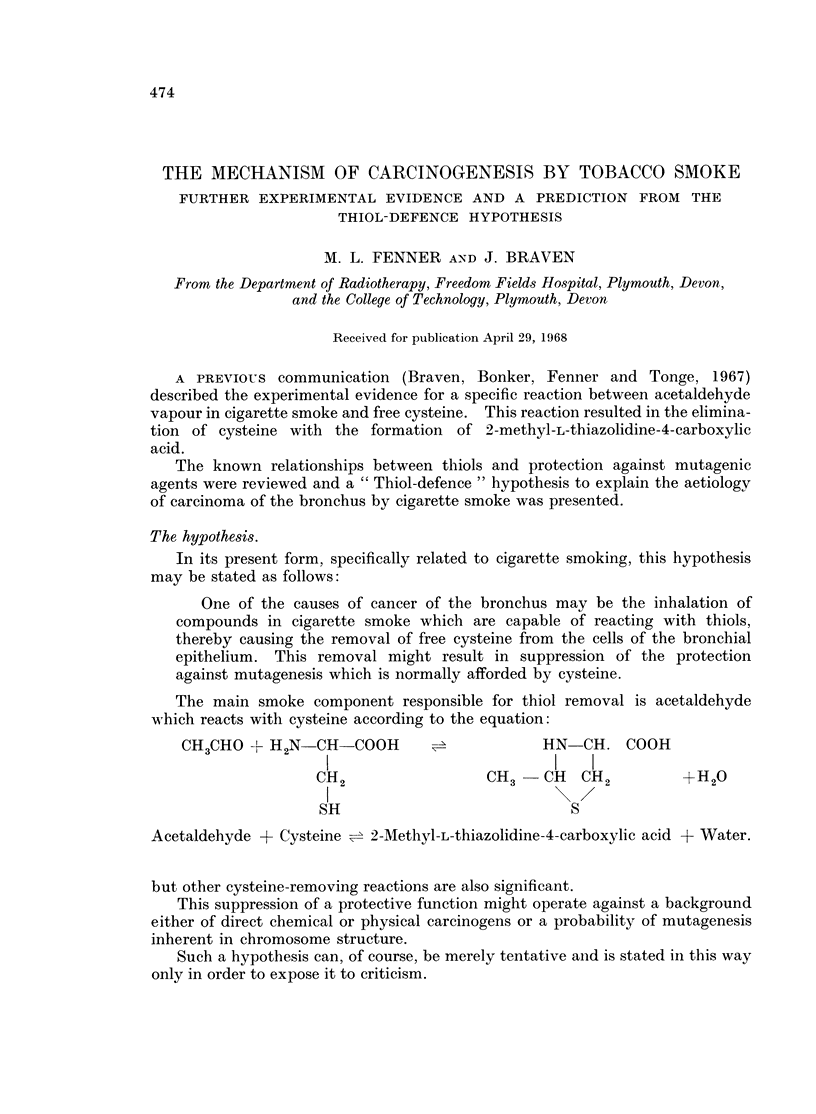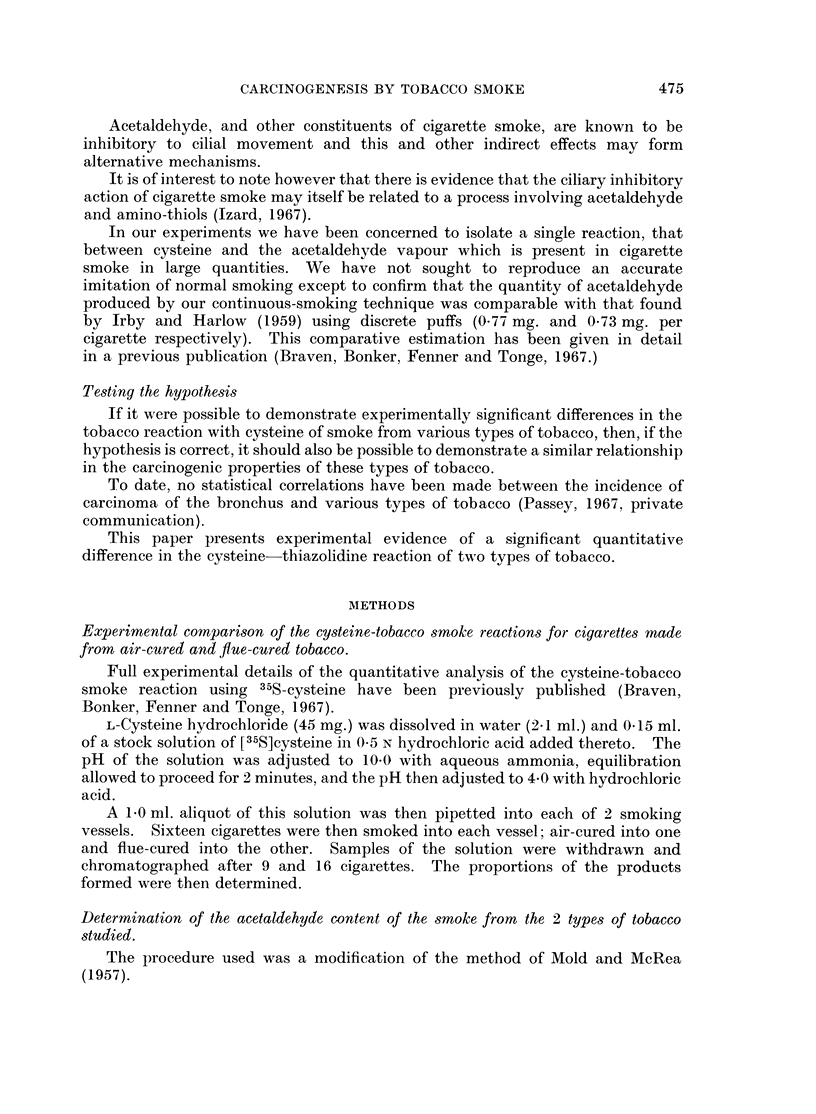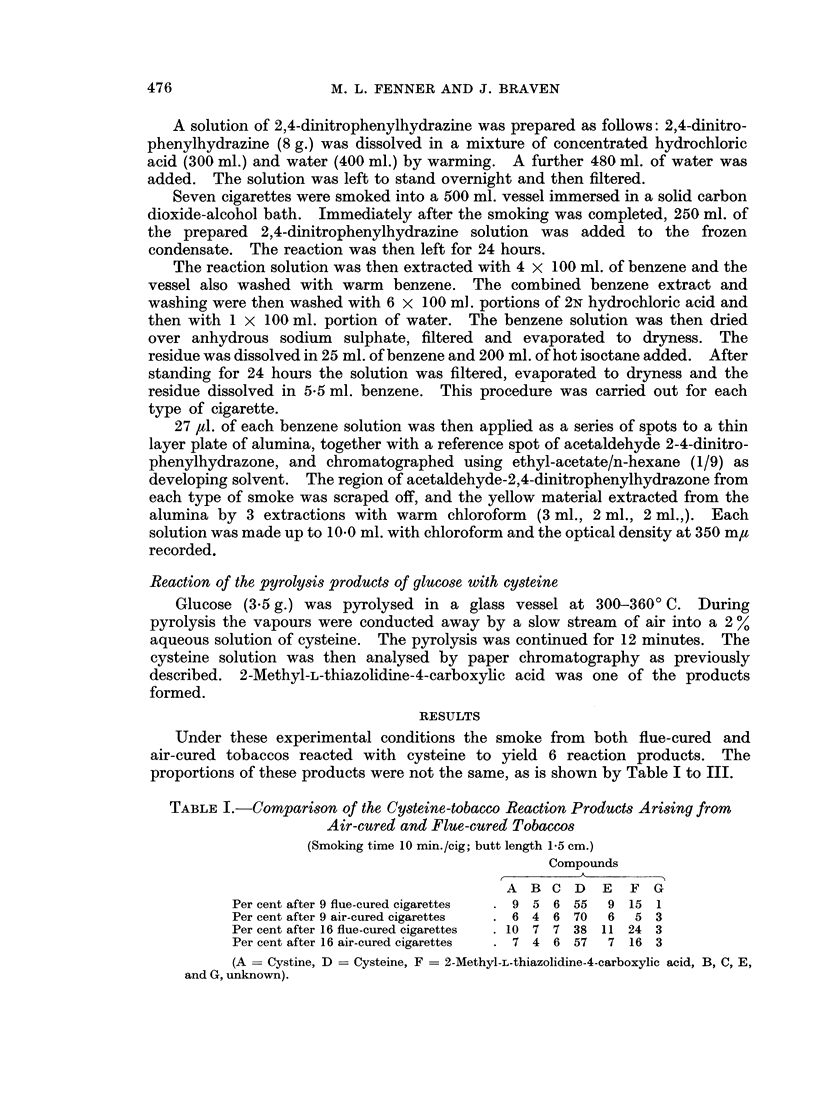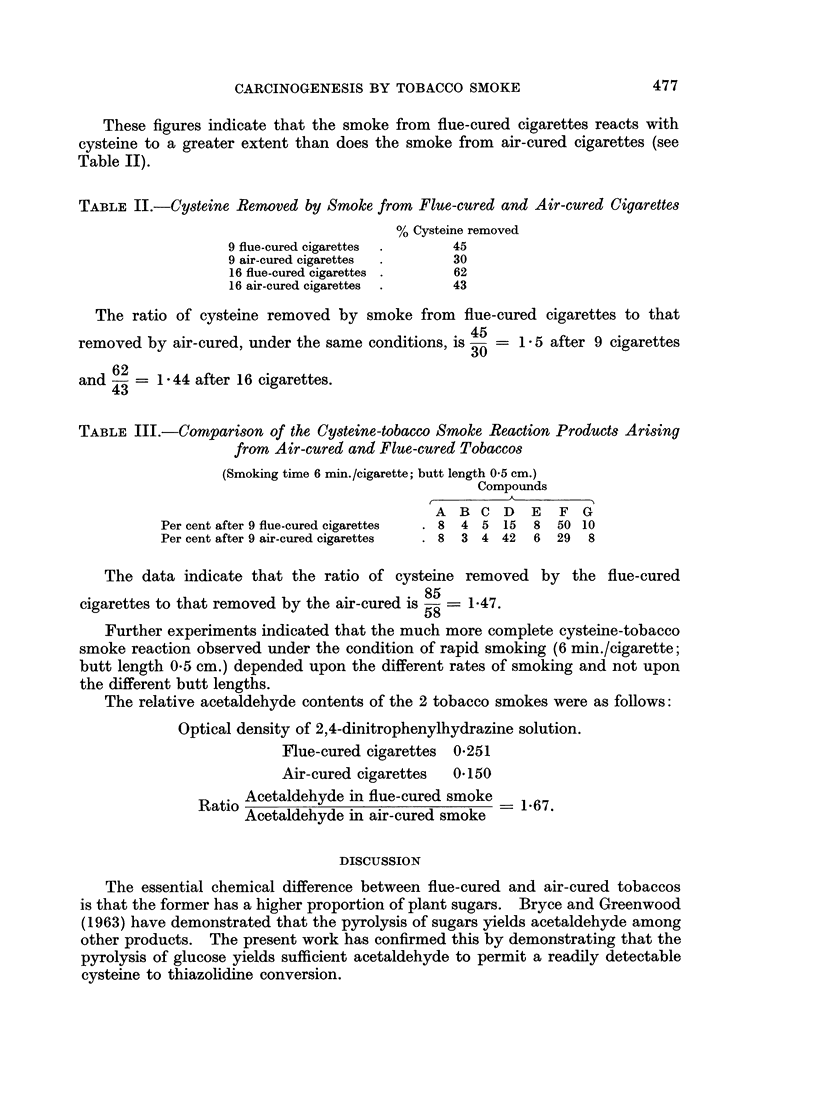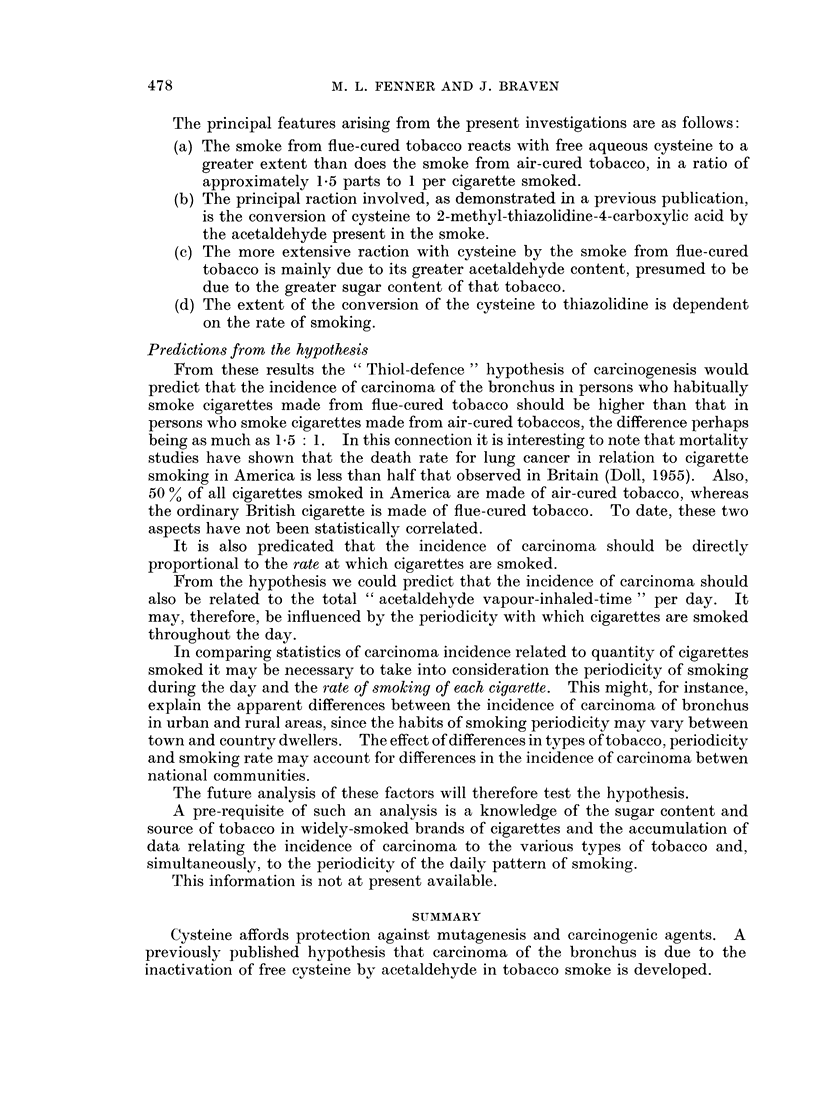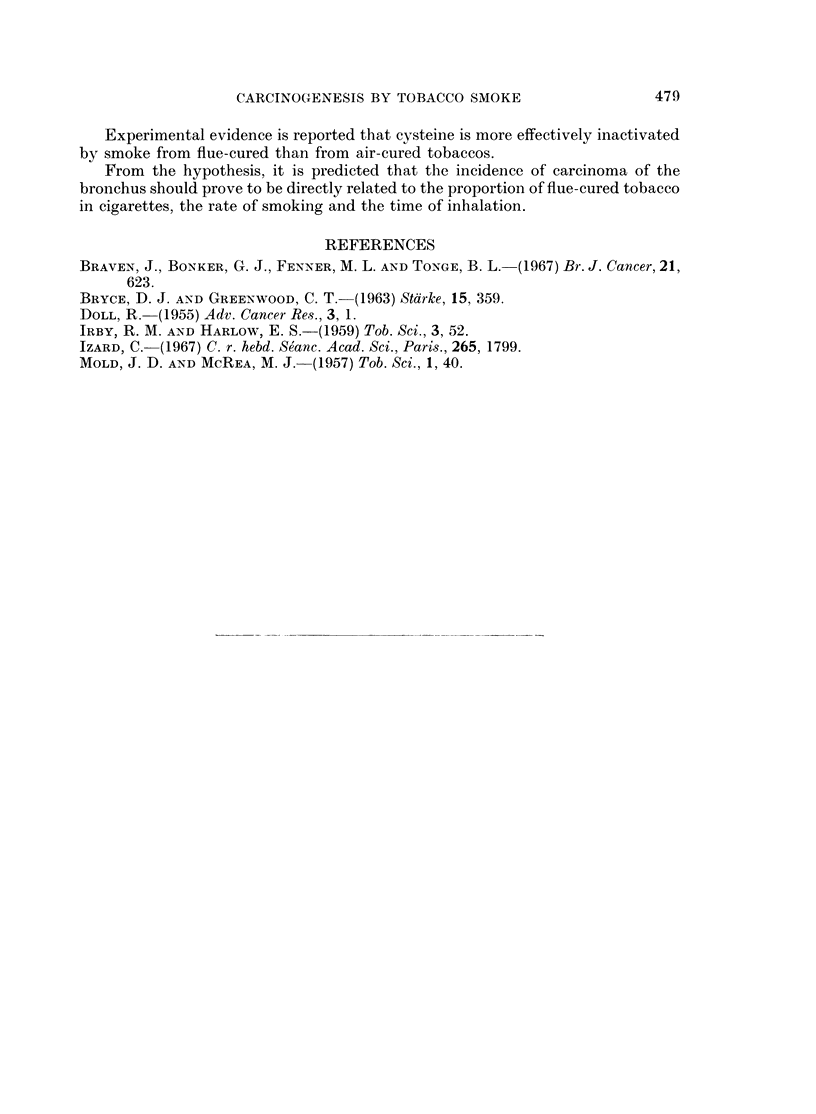# The mechanism of carcinogenesis by tobacco smoke. Further experimental evidence and a prediction from the thiol-defence hypothesis.

**DOI:** 10.1038/bjc.1968.55

**Published:** 1968-09

**Authors:** M. L. Fenner, J. Braven


### THE MECHANISM OF CARCINOGENESIS BY TOBACCO SMOKE

### FURTHER EXPERIMENTAL EVIDENCE AND A PREDICTION FROM THE THIOL-DEFENCE HYPOTHESIS

M. L. FENNER AND J. BRAVEN

*From the Department of Radiotherapy, Freedom Fields Hospital, Plymouth, Devon, and the College of Technology, Plymouth, Devon*

Received for publication April 29, 1968

A PREVIOUS communication (Braven, Bonker, Fenner and Tonge, 1967) described the experimental evidence for a specific reaction between acetaldehyde vapour in cigarette smoke and free cysteine. This reaction resulted in the elimination of cysteine with the formation of 2-methyl-L-thiazolidine-4-carboxylic acid.

The known relationships between thiols and protection against mutagenic agents were reviewed and a " Thiol-defence " hypothesis to explain the aetiology of carcinoma of the bronchus by cigarette smoke was presented.

*The hypothesis.*

In its present form, specifically related to cigarette smoking, this hypothesis may be stated as follows:

> One of the causes of cancer of the bronchus may be the inhalation of compounds in cigarette smoke which are capable of reacting with thiols, thereby causing the removal of free cysteine from the cells of the bronchial epithelium. This removal might result in suppression of the protection against mutagenesis which is normally afforded by cysteine.

The main smoke component responsible for thiol removal is acetaldehyde which reacts with cysteine according to the equation:

Acetaldehyde + Cysteine ⇌ 2-Methyl-L-thiazolidine-4-carboxylic acid + Water.

but other cysteine-removing reactions are also significant.

This suppression of a protective function might operate against a background either of direct chemical or physical carcinogens or a probability of mutagenesis inherent in chromosome structure.

Such a hypothesis can, of course, be merely tentative and is stated in this way only in order to expose it to criticism.

Acetaldehyde, and other constituents of cigarette smoke, are known to be inhibitory to cilial movement and this and other indirect effects may form alternative mechanisms.

It is of interest to note however that there is evidence that the ciliary inhibitory action of cigarette smoke may itself be related to a process involving acetaldehyde and amino-thiols (Izard, 1967).

In our experiments we have been concerned to isolate a single reaction, that between cysteine and the acetaldehyde vapour which is present in cigarette smoke in large quantities. We have not sought to reproduce an accurate imitation of normal smoking except to confirm that the quantity of acetaldehyde produced by our continuous-smoking technique was comparable with that found by Irby and Harlow (1959) using discrete puffs (0·77 mg. and 0·73 mg. per cigarette respectively). This comparative estimation has been given in detail in a previous publication (Braven, Bonker, Fenner and Tonge, 1967.)

*Testing the hypothesis*

If it were possible to demonstrate experimentally significant differences in the tobacco reaction with cysteine of smoke from various types of tobacco, then, if the hypothesis is correct, it should also be possible to demonstrate a similar relationship in the carcinogenic properties of these types of tobacco.

To date, no statistical correlations have been made between the incidence of carcinoma of the bronchus and various types of tobacco (Passey, 1967, private communication).

This paper presents experimental evidence of a significant quantitative difference in the cysteine—thiazolidine reaction of two types of tobacco.

### METHODS

*Experimental comparison of the cysteine-tobacco smoke reactions for cigarettes made from air-cured and flue-cured tobacco.*

Full experimental details of the quantitative analysis of the cysteine-tobacco smoke reaction using $^{35}$S-cysteine have been previously published (Braven, Bonker, Fenner and Tonge, 1967).

L-Cysteine hydrochloride (45 mg.) was dissolved in water (2·1 ml.) and 0·15 ml. of a stock solution of [$^{35}$S]cysteine in 0·5 N hydrochloric acid added thereto. The pH of the solution was adjusted to 10·0 with aqueous ammonia, equilibration allowed to proceed for 2 minutes, and the pH then adjusted to 4·0 with hydrochloric acid.

A 1·0 ml. aliquot of this solution was then pipetted into each of 2 smoking vessels. Sixteen cigarettes were then smoked into each vessel; air-cured into one and flue-cured into the other. Samples of the solution were withdrawn and chromatographed after 9 and 16 cigarettes. The proportions of the products formed were then determined.

*Determination of the acetaldehyde content of the smoke from the 2 types of tobacco studied.*

The procedure used was a modification of the method of Mold and McRea (1957).

A solution of 2,4-dinitrophenylhydrazine was prepared as follows: 2,4-dinitrophenylhydrazine (8 g.) was dissolved in a mixture of concentrated hydrochloric acid (300 ml.) and water (400 ml.) by warming. A further 480 ml. of water was added. The solution was left to stand overnight and then filtered.

Seven cigarettes were smoked into a 500 ml. vessel immersed in a solid carbon dioxide-alcohol bath. Immediately after the smoking was completed, 250 ml. of the prepared 2,4-dinitrophenylhydrazine solution was added to the frozen condensate. The reaction was then left for 24 hours.

The reaction solution was then extracted with $4 \times 100$ ml. of benzene and the vessel also washed with warm benzene. The combined benzene extract and washing were then washed with $6 \times 100$ ml. portions of 2N hydrochloric acid and then with $1 \times 100$ ml. portion of water. The benzene solution was then dried over anhydrous sodium sulphate, filtered and evaporated to dryness. The residue was dissolved in 25 ml. of benzene and 200 ml. of hot isoctane added. After standing for 24 hours the solution was filtered, evaporated to dryness and the residue dissolved in 5·5 ml. benzene. This procedure was carried out for each type of cigarette.

27 $\mu$l. of each benzene solution was then applied as a series of spots to a thin layer plate of alumina, together with a reference spot of acetaldehyde 2-4-dinitrophenylhydrazone, and chromatographed using ethyl-acetate/n-hexane (1/9) as developing solvent. The region of acetaldehyde-2,4-dinitrophenylhydrazone from each type of smoke was scraped off, and the yellow material extracted from the alumina by 3 extractions with warm chloroform (3 ml., 2 ml., 2 ml.,). Each solution was made up to 10·0 ml. with chloroform and the optical density at 350 m$\mu$ recorded.

*Reaction of the pyrolysis products of glucose with cysteine*

Glucose (3·5 g.) was pyrolysed in a glass vessel at 300–360° C. During pyrolysis the vapours were conducted away by a slow stream of air into a 2% aqueous solution of cysteine. The pyrolysis was continued for 12 minutes. The cysteine solution was then analysed by paper chromatography as previously described. 2-Methyl-L-thiazolidine-4-carboxylic acid was one of the products formed.

### RESULTS

Under these experimental conditions the smoke from both flue-cured and air-cured tobaccos reacted with cysteine to yield 6 reaction products. The proportions of these products were not the same, as is shown by Table I to III.

TABLE I.—*Comparison of the Cysteine-tobacco Reaction Products Arising from Air-cured and Flue-cured Tobaccos*

(Smoking time 10 min./cig; butt length 1·5 cm.)

| | Compounds | | | | | | |
|---|---|---|---|---|---|---|---|
| | A | B | C | D | E | F | G |
| Per cent after 9 flue-cured cigarettes | 9 | 5 | 6 | 55 | 9 | 15 | 1 |
| Per cent after 9 air-cured cigarettes | 6 | 4 | 6 | 70 | 6 | 5 | 3 |
| Per cent after 16 flue-cured cigarettes | 10 | 7 | 7 | 38 | 11 | 24 | 3 |
| Per cent after 16 air-cured cigarettes | 7 | 4 | 6 | 57 | 7 | 16 | 3 |

(A = Cystine, D = Cysteine, F = 2-Methyl-L-thiazolidine-4-carboxylic acid, B, C, E, and G, unknown).

These figures indicate that the smoke from flue-cured cigarettes reacts with cysteine to a greater extent than does the smoke from air-cured cigarettes (see Table II).

TABLE II.—*Cysteine Removed by Smoke from Flue-cured and Air-cured Cigarettes*

| | % Cysteine removed |
|---|---|
| 9 flue-cured cigarettes . | 45 |
| 9 air-cured cigarettes . | 30 |
| 16 flue-cured cigarettes . | 62 |
| 16 air-cured cigarettes . | 43 |

The ratio of cysteine removed by smoke from flue-cured cigarettes to that removed by air-cured, under the same conditions, is $\frac{45}{30} = 1 \cdot 5$ after 9 cigarettes and $\frac{62}{43} = 1 \cdot 44$ after 16 cigarettes.

TABLE III.—*Comparison of the Cysteine-tobacco Smoke Reaction Products Arising from Air-cured and Flue-cured Tobaccos*

(Smoking time 6 min./cigarette; butt length 0·5 cm.)

| | Compounds | | | | | | |
|---|---|---|---|---|---|---|---|
| | A | B | C | D | E | F | G |
| Per cent after 9 flue-cured cigarettes . | 8 | 4 | 5 | 15 | 8 | 50 | 10 |
| Per cent after 9 air-cured cigarettes . | 8 | 3 | 4 | 42 | 6 | 29 | 8 |

The data indicate that the ratio of cysteine removed by the flue-cured cigarettes to that removed by the air-cured is $\frac{85}{58} = 1{\cdot}47$.

Further experiments indicated that the much more complete cysteine-tobacco smoke reaction observed under the condition of rapid smoking (6 min./cigarette; butt length 0·5 cm.) depended upon the different rates of smoking and not upon the different butt lengths.

The relative acetaldehyde contents of the 2 tobacco smokes were as follows:

Optical density of 2,4-dinitrophenylhydrazine solution.

| | |
|---|---|
| Flue-cured cigarettes | 0·251 |
| Air-cured cigarettes | 0·150 |

$$\text{Ratio } \frac{\text{Acetaldehyde in flue-cured smoke}}{\text{Acetaldehyde in air-cured smoke}} = 1{\cdot}67.$$

### DISCUSSION

The essential chemical difference between flue-cured and air-cured tobaccos is that the former has a higher proportion of plant sugars. Bryce and Greenwood (1963) have demonstrated that the pyrolysis of sugars yields acetaldehyde among other products. The present work has confirmed this by demonstrating that the pyrolysis of glucose yields sufficient acetaldehyde to permit a readily detectable cysteine to thiazolidine conversion.

The principal features arising from the present investigations are as follows:

(a) The smoke from flue-cured tobacco reacts with free aqueous cysteine to a greater extent than does the smoke from air-cured tobacco, in a ratio of approximately 1·5 parts to 1 per cigarette smoked.
(b) The principal raction involved, as demonstrated in a previous publication, is the conversion of cysteine to 2-methyl-thiazolidine-4-carboxylic acid by the acetaldehyde present in the smoke.
(c) The more extensive raction with cysteine by the smoke from flue-cured tobacco is mainly due to its greater acetaldehyde content, presumed to be due to the greater sugar content of that tobacco.
(d) The extent of the conversion of the cysteine to thiazolidine is dependent on the rate of smoking.

*Predictions from the hypothesis*

From these results the " Thiol-defence " hypothesis of carcinogenesis would predict that the incidence of carcinoma of the bronchus in persons who habitually smoke cigarettes made from flue-cured tobacco should be higher than that in persons who smoke cigarettes made from air-cured tobaccos, the difference perhaps being as much as 1·5 : 1. In this connection it is interesting to note that mortality studies have shown that the death rate for lung cancer in relation to cigarette smoking in America is less than half that observed in Britain (Doll, 1955). Also, 50 % of all cigarettes smoked in America are made of air-cured tobacco, whereas the ordinary British cigarette is made of flue-cured tobacco. To date, these two aspects have not been statistically correlated.

It is also predicated that the incidence of carcinoma should be directly proportional to the *rate* at which cigarettes are smoked.

From the hypothesis we could predict that the incidence of carcinoma should also be related to the total " acetaldehyde vapour-inhaled-time " per day. It may, therefore, be influenced by the periodicity with which cigarettes are smoked throughout the day.

In comparing statistics of carcinoma incidence related to quantity of cigarettes smoked it may be necessary to take into consideration the periodicity of smoking during the day and the *rate of smoking of each cigarette*. This might, for instance, explain the apparent differences between the incidence of carcinoma of bronchus in urban and rural areas, since the habits of smoking periodicity may vary between town and country dwellers. The effect of differences in types of tobacco, periodicity and smoking rate may account for differences in the incidence of carcinoma betwen national communities.

The future analysis of these factors will therefore test the hypothesis.

A pre-requisite of such an analysis is a knowledge of the sugar content and source of tobacco in widely-smoked brands of cigarettes and the accumulation of data relating the incidence of carcinoma to the various types of tobacco and, simultaneously, to the periodicity of the daily pattern of smoking.

This information is not at present available.

SUMMARY

Cysteine affords protection against mutagenesis and carcinogenic agents. A previously published hypothesis that carcinoma of the bronchus is due to the inactivation of free cysteine by acetaldehyde in tobacco smoke is developed.

Experimental evidence is reported that cysteine is more effectively inactivated by smoke from flue-cured than from air-cured tobaccos.

From the hypothesis, it is predicted that the incidence of carcinoma of the bronchus should prove to be directly related to the proportion of flue-cured tobacco in cigarettes, the rate of smoking and the time of inhalation.

### REFERENCES

Braven, J., Bonker, G. J., Fenner, M. L. and Tonge, B. L.—(1967) *Br. J. Cancer*, **21**, 623.

Bryce, D. J. and Greenwood, C. T.—(1963) *Stärke*, **15**, 359.

Doll, R.—(1955) *Adv. Cancer Res.*, **3**, 1.

Irby, R. M. and Harlow, E. S.—(1959) *Tob. Sci.*, **3**, 52.

Izard, C.—(1967) *C. r. hebd. Séanc. Acad. Sci., Paris.*, **265**, 1799.

Mold, J. D. and McRea, M. J.—(1957) *Tob. Sci.*, **1**, 40.